# Improving Security of Web Servers in Critical IoT Systems through Self-Monitoring of Vulnerabilities

**DOI:** 10.3390/s22135004

**Published:** 2022-07-02

**Authors:** Linxuan Song, Marisol García-Valls

**Affiliations:** 1 Beijing University of Posts and Telecommunications, Beijing 100876, China; 2018213147@bupt.cn; 2 Universitat Politècnica de València, 46022 Valencia, Spain

**Keywords:** security, web technology, web servers, web programming, vulnerability detection, self monitoring, IoT, IIoT, Cyber Physical Sytems, critical systems

## Abstract

IoT (Internet of Things) systems are complex ones that may comprise large numbers of sensing and actuating devices; and servers that store data and further configure the operation of such devices. Usually, these systems involve real-time operation as they are closely bound to particular physical processes. This real-time operation is often threatened by the security solutions that are put in place to alleviate the ever growing attack surface in IoT. This paper focuses on critical IoT domains where less attention has been paid to the web security aspects. The main reason is that, up to quite recently, web technologies have been considered unreliable and had to be avoided by design in critical systems. In this work, we focus on the server side and on how attacks propagate from server to client as vulnerabilities and from client to unprotected servers; we describe the concerns and vulnerabilities introduced by the intensive usage of web interfaces in IoT from the server templating engines perspective. In this context, we propose an approach to perform self monitoring on the server side, propagating the self monitoring to the IoT system devices; the aim is to provide rapid detection of security vulnerabilities with a low overhead that is transparent to the server normal operation. This approach improves the control over the vulnerability detection. We show a set of experiments that validate the feasibility of our approach.

## 1. Introduction

The IoT paradigm is a key step in the way that modern systems are conceived and architected. The union of smart devices and servers connected to a potential myriad of sensing and actuating nodes constitutes a powerful infrastructure to develop applications and systems that enhance the intelligence and capacities of the human users. This spans from eHealth [1], to smart manufacturing [2] and automation, smart cities, and many more.

The core concept at the heart of IoT has been the ultra-fast end-to-end interconnection across all devices, built on the advances in networks such as 5G technology and the new conception of 6G. The integration with other ICT paradigms and technologies like Cloud Computing, Fog Computing, DevOps, and software frameworks have provided a set of tools to build unprecedented applications [3].

Today, most IoT systems are either intensive in the usage of web interactions or, at least, have some part that uses web protocols and tools. Not only the usability of these systems and applications, but also the communication across devices is based on web technologies and web protocols thanks to the W3C [4] standards. Web interactions rely on HTTP [5] and HTTPS [6] protocols and the REST [7] scheme. Additionally, most IoT platforms are built on a web interface that acts as a centralized dashboard and a unified managing server to communicate with the system devices.

Why IoT systems security is increasingly compromised is inherent to their nature. They are composed of large numbers of heterogeneous devices like sensors, actuators, computer nodes, and servers; they are highly interconnected (which includes Internet access) and rely on software platforms, mostly developed as accidental systems. On top of this, incorporating web technologies has enlarged the attack surface for cyber criminals.

Existing approaches to improving security in IoT focus on a subset of the components that these systems contain, mostly related to cryptography, networking transmission, routing, etc. The software security side has also been researched with code analysis techniques, operating system security, forensics, etc. This is not sufficient in critical IoT subsystems as an error may have catastrophic consequences. However, there is a difficult equilibrium: on the one hand, critical IoT systems need to provide safety guarantees therefore reducing their exposure to vulnerabilties, but, on the other hand, IoT servers may need to intensively use web technologies, software frameworks, and libraries that allow the developer to build functionality that would not be possible otherwise.

In this work, we provide an approach to improve the security of the web servers acting as entry points to a critical IoT; we design a light middleware that actively performs self monitoring of the whole system and allows the system operators to launch tests that detect vulnerabilities in the nodes. This contributes to raising awareness about particular vulnerabilities of some accidental IoT software constituents, e.g., platforms such as Python Flask, Node.js, etc., to detect and even prevent the main vulnerabilities that may be exposed.

In this approach, servers have two main functions. First, they monitor and control a number of IoT devices guided by the action of a remote operator. Second, servers provide the front-end to the clients (i.e., to the operators). The security is analysed from the perspective of prevention of injection attacks for a particular development platform using a specific templating engine. The server incorporates an additional logic and functionality that allows for actively self-monitoring injection vulnerabilities. In case some vulnerability is detected, the server triggers an alarm and provides recommendations on how to eliminate it. We validate the execution of our software by analysing the performance of the system to ensure that it is not degraded by the addition of the security self-monitoring functionality.

### 1.1. Terminology and Threats in Web Security

A *threat* is any possible danger that can exploit a vulnerability, damage or destroy an asset intentionally or accidentally. According to [8], a threat is a potential for security violation; this includes unauthorized access or denial of service. In a manufacturing system, an asset can range from some collected data reading, to a software piece, or  to a physical device impact. Threats may negatively affect the security of the systems; they exist constantly regardless of whether they will succeed or not in the end.

A *vulnerability* is the weakness or flaw in a system that reduces its security. The key difference with respect to a threat is that a vulnerability does exist in the system while a threat probably does. A vulnerability can exist in any part of the system (for example, hardware, software, network, server, etc.) and can be exploited by an external force like an attacker, a script, or some tools.

A *risk* is the potential that a threat can exploit a vulnerability to damage the assets in the system. It can be seen as the combined realization of the two concepts above: threat and vulnerability. According to ISO/IEC 27005 [9], a risk in Information Technology can be measured by the combination of the probability of occurrence of an event and its consequence.

Main bodies like the International Organization for Standardization (ISO) 27001 have listed the requirements to implement and improve the security settings for web applications [10]. The top ten most critical security risks [11] are regularly published for the companies and developers to raise the awareness of web application security in order to reduce the damage caused by potential vulnerabilities of applications. However, accordingly, there are recommended programming patterns that help the users to repair the unsafe configurations and minimize the risks [12].

A selected set of *security risks for web interfaces* are listed below:

- *Broken access control*. Access control forces users to act strictly inside the limits of the application area to which they have been granted permission. If the applied access control policy is not successful for a given user, s/he will have access to valuable resources that should be unreachable to her/him.
- *Cryptographic failures* yield (sensitive) *data exposure*. For this reason, data encryption should be kept to the newest standards, avoiding that systems (browser, data base, etc.) use deprecated cryptographic functions to encrypt data, passwords, etc. Classifying the security level of all the system data is also necessary to determine what cryptographic algorithms to use and how to certify and validate the receiver.
- *Injection* includes a number of risks like, e.g., *cross-site scripting* (XSS) and *SQL injection*. An injection occurs when the user input is not properly validated and filtered, and it becomes part of either the web page or the data base; if a malicious input is directly used by the application, hostile data may be injected to the application records or database, causing a longer lasting damage to the system. This can be partly fixed by separating commands from data and by input validation through proper filters; by using safer APIs and SQL controls that limit the unauthorized operations and avoid disclosure of sensitive data; and by reviewing the source code.
- *Security misconfiguration* is often introduced by enabling, disabling, installing, or deinstalling features; leaving a default account and password unchanged; using outdated components; and not enabling the latest security functions.
- *Identification and authentication failures* are usually the result of brute-force attacks and/or cryptographic failures, by using scripts that repeatedly attempt the validation of combinations of usernames and passwords automatically. Such actions are known as brute force attacks. Such risks are present when there is a credentials database with weak secure authentication (e.g., *admin* user with password *abc123*); or when attackers take advantage of leaked session IDs to gain control over a communication. More steps for verification (e.g., code, secure questions, using other devices such the mobile, face recognition, etc.) can reduce the risk of brute force attacks. Other protective measures are to use complex passwords and remove simple test accounts.
- *Software and data integrity failures* are likely to take place when the application depends on untrusted libraries, modules, and/or other resources.
- *Security logging and monitoring failures* has to be a constant task to detect active attacks. Creating logs of failed login attempts is a basic strategy to prevent it; however, it may not be sufficient for several reasons: some failed login attempts may not be recorded; in addition, warnings and errors may not provide a clear picture of the situation. The developer should ensure that: (1) all the access control and failures can be recorded with sufficient user data, (2) the logged data are encrypted in case of injection, and (3) establish an error report mechanism.
- *Server-side request forgery* happens when an application fetches a remote resource without validating the URL submitted by the user. As a result, the attacker can send a crafted request to unexpected links even if the application is protected by firewalls or VPN. The application side has to avoid this by filtering and validating all data submitted by the user, set a series of URL schemas, and avoid sending raw responses to the client.

### 1.2. Related Work

Real-time systems, embedded systems, wireless sensor networks, and control systems were the precursors of Cyber-Physical Systems (CPS) [13] and IoT. In such domains, web technologies and middleware in general were disregarded as they were considered to introduce unpredictable behavior yielding to non-stable execution times.

Since the spread of the IoT paradigm, web technologies have progressively gained territory because they are unbeatable in providing a highly accessible collaborative platform; they include thin clients that can access the system from anywhere, anytime, and with solely the need of a web front-end.

Nevertheless, bringing web technologies into IoT systems enhances the attack surface for criminals [14]; and this is still dangerous in critical IoT domains like eHealth, critical infrastructures, avionics, etc. This requires that the traditional functional focus of web applications is changed. Years ago, the focus was primarily on the functional development, and this caused a dramatic increase of successful attacks over the last few years. The white paper of [15] shows the level of penetration of [16], the software-related vulnerabilities into the critical IoT domain, and how this weakens the overall operation process if appropriate measures are not put in place.

Some web platforms have become useless for this purpose, and others have increased their security over the last few years to avoid attacks. As new IoT platforms have added a number of functions for security, the Internet has also transitioned to security protocols such as HTTPS [6] and TLS [17], among others. At the same time, other technologies have appeared to provide real-time web interaction.

Platforms like [18], Datadog, Azure IoT, and many other similar initiatives are web-based, supporting monitoring of the smart IoT devices in real-time using real-time web technology.

Now, operators can access the IoT devices from anywhere through a web interface. Because the browser is typically used as a combined display and communication end-point for operators (clients) to interact with the server, there is a risk that cybercriminals attempt to use the same interface to penetrate the system. For this reason, many cyber attacks use the browser client as an entry point to the system.

Although some general purpose security guidelines for web based systems have appeared such as usage of end-to-end encrypted channels [6,17], certificate management [19], usage of appropriate browser policies [20,21], and many others; there is a lack of detailed analysis of particular software platforms used to develop servers. Collaborative and open source frameworks such as SonarQube have also been enhanced to support the improvement of the safety properties of the software [22] but still have not merged web interfacing in critical IoT software.

Given the recent push of artificial intelligence and machine learning, a number of contributions on web security have appeared in these domains. Some examples of these are Ref. [23] that provides an intrusion identification scheme based on a convolutional neural network to improve the accuracy of intrusion detection; in addition, Ref. [24] explains an approach based on deep learning for the detection of different cyber attacks in the web of things (WoT) focusing on prevention of DoS (Denial of Service attacks), U2R (User to Root) attacks, or R2L (Remote to user); these are based on detection of packets’ characteristics and other techniques focusing on networking security. Therefore, this type of contribution is quite different from ours as we focus on the application and web programming level that is not comparable to the pure network layer.

In comparison to the large amount of networking security contributions, a significantly more reduced proportion of works have targeted web security at the application level. Precisely, Ref. [25] compiles a list of web security criteria for tools that implement web scanning. Ref. [26] provides a mechanism to recognize exploitable vulnerabilities but strictly focused on websites implemented with web-content management systems. In [27], authors provide a mechanism and tool for port scanning of web servers combined with cross-site scripting and SQL injection; but this contribution is limited to a pure functional perspective  [28], offering a simulated exercise of cross-site scripting attacks on simulated browsers. Ref. [29] exemplifies automated vulnerability checking but restricted to network servers on the basis of the pure networking traffic characteristics. Ref. [30] emphasizes the importance of proper notifications over web vulnerabilities; from this work, we can derive that the integration of the scanning methods in the web application is most effective for controlling and reporting vulnerabilities. Ref. [31] provides a scanning technique for detecting IoT devices with vulnerabilities, concentrating on IP generation to launch remote checks based on techniques similar to ZMAP [32] or Shodan [33].

In summary, our paper intends to contribute at the application level by providing an approach to improve the security of IoT servers that use mainstream software platforms; the idea is that the server is capable of self-monitoring vulnerabilities that are integrated in the system and provide an alert to solve it. Instead of using general purpose web scanners, this approach is based on integrating the scanning mechanism inside the application to achieve two main advantages. On the one hand, it improves the chances of finding vulnerabilities for the particular web technologies used by the server and devices. On the other hand, in domains requiring more control over the whole system operation such as critical systems, this approach provides higher control over which vulnerabilities are searched for and also allows the development team to integrate vulnerability checks as new issues are discovered. The main differences with respect to existing works on web security scanning is precisely the integration inside the application, the actual implementation integrated inside the web server, and the specifics added for Python servers, e.g., by the detection of template injection attacks when a templating engine must be used.

### 1.3. Paper Organization

The rest of the paper is structured as follows. Section 2 describes the baseline material and technologies like the assumed threat model, the system requirements imposed as derived from it, and the vulnerabilities that are considered. Section 3 describes the proposed framework for self-monitoring that includes the IoT system model and device model, the server design, the potential vulnerabilities that the specific server technology adds, and the design of the self-monitoring activity. Section 4 shows the validation of our approach, describing the experiments carried out and the analysis of the server response times. Section 5 concludes the paper.

## 2. Baseline

### 2.1. Threat Model and System Requirements

The IoT edge nodes are subject to having hardware and software vulnerabilities. Nevertheless, this paper targets the software vulnerabilities that can stem from different sources:

- *Server*. Attacks may be triggered by the vulnerabilities introduced by the usage of particular software platforms for programming the backend logic. Examples of such platforms are Node.js, Flask, Django, or PHP, among others.
- *IoT sensor nodes*. Attacks may come from the installed software code (or by the particular version of a software), by intercepting the unencrypted network traffic over a wireless channel, etc.
- *Personal device*. These are often inconvenient access nodes as they combine extensive software (including apps) and wireless communications and an intensive human usage. Here, attacks may be raised by different sources:
  - the browser code as the mobile phone may access some insecure URL and download malicious code, or contact an illegitimate server via HTTP;
  - the installed software. The user may have installed insecure Apps. There are a number of apps that contain well known vulnerabilities that can be exploited; For example, some App may use an underlying HTTP communication, or may have some permissions provided by an unconscious user that allow it to access user private data;
  - human error or misconduct. Critical IoT systems typically implement protocols for safety assurance that include the human factor. Nevertheless, human behavior is always accounted for some level of error either intentional or not;
- *Interactions across devices*. There must be a well-modeled and thought interaction protocol among all devices that comprises the security privileges of nodes, their allowed interactions and functionality, as well as the access level rights to data and information. Moreover, interactions should happen over a safe communication channel that relies on integrity, confidentiality, and authenticity; this requires encryption, authentication, access rights, and secure programming are put properly in place.

The system requirements are listed below:

- *Automated self-monitoring of vulnerabilities*. The server must contain the logic to perform fully autonomous monitoring of its security status with respect to different types of vulnerabilities, e.g., injection;
- *On demand security checks*. The authorized system operators can select a specific IoT device to check, including the server node;
- *Provide access level privileges for operators*. Not all clients/operators can access the security logs and status logs of the devices produced by the server as a result of the online checks;
- *Modular design including flexible testing logic*. The server software can be updated dynamically to enhance the number and type of tests performed on the system nodes. Additionally, a modular design provides higher flexibility and improves maintainability.

Here, we focus on the security issues that are raised in such an environment. Applying the suitable programming techniques are of paramount importance given the myriad of existing software libraries, languages, constant software updates, functional upgrading, etc.

### 2.2. Vulnerabilitites

In this work, the analysed vulnerabilities are relative to malicious content *injection*. These are explained below.

*Cross-site scripting (XSS)*. It is the most popular injection. It occurs when malicious contents like scripts are submitted by the user or attacker and injected to the server side. According to the place of injection, there are three types of XSS attacks: reflected, stored, and DOM-Based XSS. Only the first two can happen to the server side, whereas the third one only makes changes to the client front-end. This makes it less interesting in our context.

For the stored one, the malicious content can be injected to the server side by inputting scripts in some location that results in the storage of the user input. Examples of such locations are mostly text input fields like those requesting the user name. There is a critical side to this input of malicious scripting if it is fed to the database: every time the user opens the website, the malicious content will be executed.

For the reflected one, the attacker can add the malicious content to a URL and send the fake link to the users via e-mails, messages or some websites. When the user opens the link and submits the specially crafted contents, the malicious script can steal the input and call particular interfaces to attack the server.

*Server-side template injection*. It occurs in a similar way with respect to the reflected XSS though only attacking the server side. Templating engines allow the developer to transmit data between the server logic and HTML codes through a set of parameterizable constructs known as the template language.

Typically, the templating engine provides methods allowing the server to directly write HTML in the form of a string—for example, function render_template_string() in Jinja2 that is often used in Python servers. If a template injection occurs at the server, malicious content will become part of such a string parameter that contains HTML. Moreover, the attacker can steal sensitive data like SESSION_KEY information by injecting particular scripts to the original URL.

*NoSQL Injection* Unlike relational databases, non-SQL data bases do not require relational tables or data formats. Instead, they provide less restrictions and consistency checks, offering better performance and scaling facilities. Nevertheless, they can be attacked from a program built in a procedural language. For example, in the case of MongoDB, this vulnerability is caused by the usage of the *$where* operator, used typically as a filter to select by particular data attributes. However, it can also execute JavaScript code. As a result, it is not recommended to use this operator as the attacker can inject malicious script code to the query statements and manipulate the database content.

## 3. Approach

### 3.1. IoT Application

**Figure 1:**
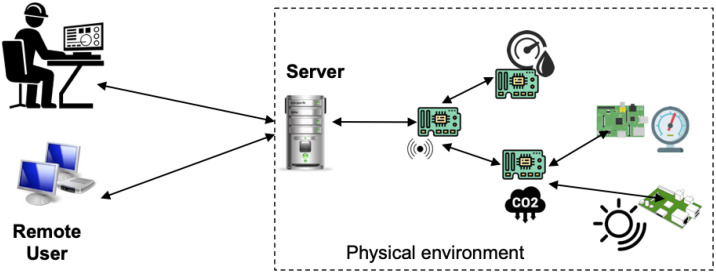
System overview.

The approach is exemplified on an IoT application system comprising a server (that could also be a reduced set of servers) that are connected to the operational physical network of IoT devices, and to a data base that stores the logs about the environment situation. Operators interact with the server to retrieve information about the facility and its associated variables (e.g., temperature, humidity, pressure, etc.); and to set the values of these actuation devices when needed. Figure 1 shows the target domain.

This smart building monitors the physical conditions of the space and adjusts actuation values to keep the comfort of occupants. In this situation, we use air conditioning devices in different rooms. The server and the devices are paired either by WiFi or Bluetooth according to the distance. There is a router that enables the communication between the devices and the server; the server will process requests and send commands if needed. JSON and MongoDB are used to store the basic settings of the devices and the operator information.

**Figure 2:**
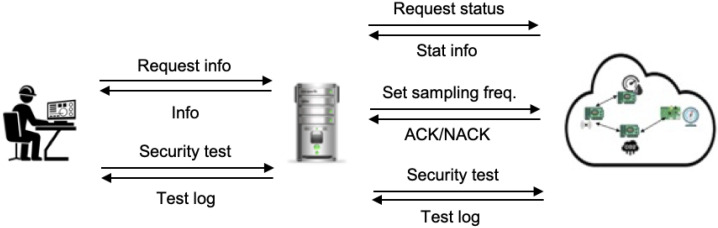
Interaction across nodes.

Python platforms like Flask are powerful (yet small) application engines that allow the developer to create the server logic in an IoT system. The server receives incoming triggers from the operator in the form of HTTP requests (see Figure 2). Requests are processed and served as indicated by the operator. If the operator requests a particular resource, the server will provide it by means of an HTTP response to the requester that includes the particular resource.

### 3.2. IoT Device Model

The modeled entities are either devices or networks. These are modeled as JSON objects comprising a number of different attributes.

The following attributes are part of an IoT device model. Attribute name is the identification code of the particular device; category indicates whether it is a *sensor*, *smart node*, *actuator*, *server*, *gateway*, etc. Attribute net contains the network or networks that the device is connected to (e.g., a particular wifi, a bluetooth connection, etc.). If the device is connected to multiple networks, a JSON array object is used that contains the identification of those networks. Attribute model provides more information about the particular subtype, vendor model, etc., of the device. Attribute position refers to the specific space (e.g., room, area, etc.) where the device is located in.

Additionally, modeling a network has the attribute name, which is the identification code of that precise network; and category, which informs about the type of network protocol that is used, e.g., WiFi, Bluetooth, etc.

An example of the above is shown in Listing 1.

**Listing 1:** Declaration of devices.

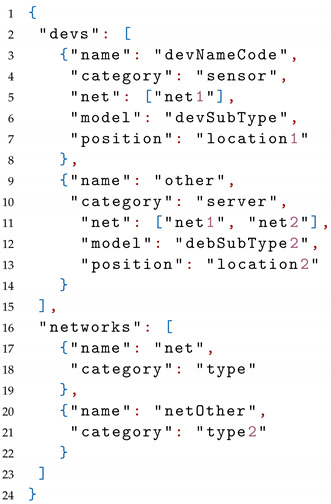

### 3.3. Server Design

The server functionality is provided to the operator and IoT devices via *services*. Services are implemented through route invocations. For example, the access to a given IoT device is defined by the Flask route instruction @app.route(’/<username>/dev’), where <username> is the identification of requesting the operator and dev is the identification code of the target device; statement name = f’username’ is used to extract the value of the provided parameter to name for future use, f being a formatted string literal.

**Figure 3:**
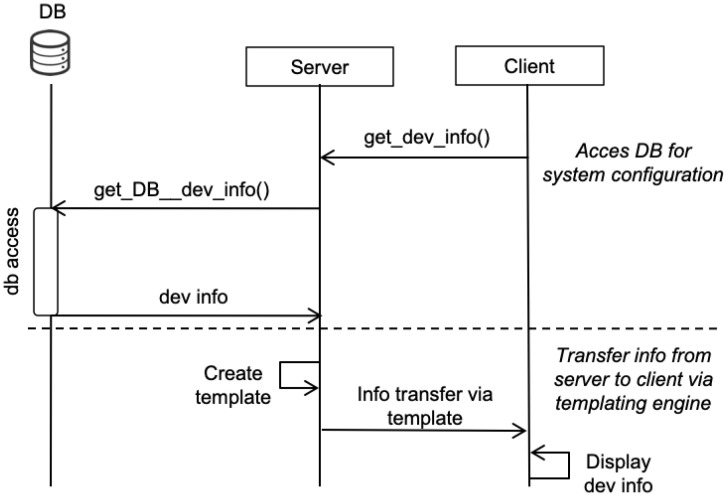
Interaction sequence.

The server stores the IoT device data in JSON format in persistent storage; the corresponding file is imported by the DATA BASE module; to do this, the following code is used with open("dbsys.json","r",encoding="utf-8") as f:data=json.loads(f.read()) that retrieves the system database in variable data, which is of Python type dict (dictionary). The interaction flow of the server with the data base (DB) and client side is shown in Figure 3.

The transfer of system data to the client side is done by the server using a templating engine.

On the client side, the data are received and processed by using a combination of HTML and templating. Listing 2 shows how this compilation and display of the device information data are done at the client side. The text URLTEXT will be replaced by the particular domain of the server and port, e.g., 127.0.0.1:5001 if the server is running on the local machine.

**Listing 2:** Server uses a templating engine to display device information at the client.

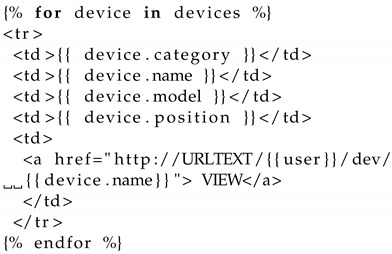

### 3.4. Potential Vulnerabilities

Flask is a Python micro framework that comprises a web environment to build web applications. The term “micro” means that there are a number of facilities left out as it is meant for fast development and light runtime that is very convenient for light IoT deployments. Aspects such no form validation are not present; however there is a lively and great community support to investigate, report, and solve security issues. There is nothing very distinct from most web frameworks, where security is something to consider carefully as there may be a number of vulnerabilities introduced by default.

An example, let us see Listing 3, which is a simple form for requesting user input that is later posted in some form to the server.

**Listing 3:** Form request.

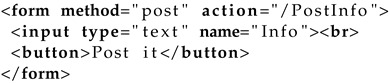

A simple Jinja2 template that renders the information provided by users is shown in Listing 4.

**Listing 4:** Data from the form is used on the server to produce a client interface.

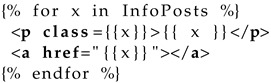

The server code that handles the form data and uses the above templating of Listing 4 is shown in Listing 5.

**Listing 5:** Form request.

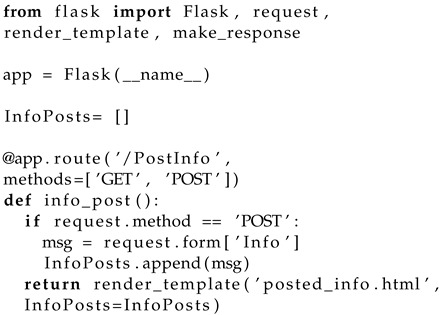

Another problematic situation is derived from the usage of the requesting URL to extract information from the server: (1) that is part of the server main memory that is not properly hidden, or (2) from the execution environment.

The first type is due to the usage of global memory space in the server in combination with templates that access global memory space, and in combination with the improper processing of a variable sent as a string in the URL string (see Listing 6).

**Listing 6:** Form request.

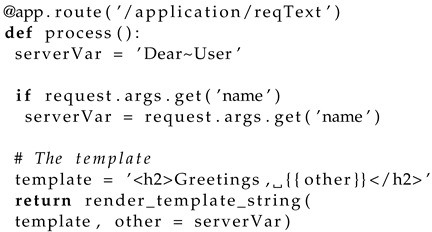

A variable name inside the URL can be extracted and later passed to a template with {{ other }}. Inside the template, this causes HTML to escape all characters automatically by default. Thus, what came inside name as part of the URL route will show in the template response. As a result, if an attacker feeds a script accessing some server environment data like {{config.items()}} or <script>alert("text")</script>, this will result in the server running the associated code.

This vulnerability can be catastrophic because it can expose sensible data. For example, if the string "?name={{person.password}}" is appended, then the access credentials can be stolen.

Furthermore, if an attacker injects other sensitive commands such as "?name={{name.config.items()}}", a large number of global properties of the systems can be exposed, e.g., SECRET_KEY.

Moreover, an attacker can also gain an advantage on this vulnerability to read, modify, or create server files and load scripts to the server.

### 3.5. Self-Monitoring Design

For checking the security of web interfaces, most test methods are based on adding payloads to the URL as input data inserted in the requests to the server. Our approach uses this scheme as the basis, complemented with an additional middleware on the server that performs self-monitoring. It is based on an additional module named *TEST* that is parameterized with two values: (1) the URL and (2) the particular test id. In addition, a *CHECK* module is implemented to control the test execution flow as follows:

1. It finds payloads suitable for the test to be run;
2. It processes the URL and conveniently crafts it with the malicious payloads.

Lastly, an INTERFACE module has been designed for managing the requests from clients/operators. This provides the entry point to the system.

**Figure 4:**
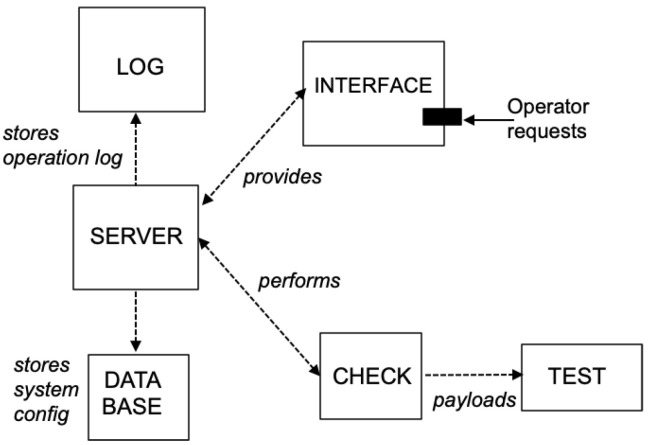
Software functions on the server side concerning testing.

Figure 4 shows the workflow among the different modules of the server that relate to the security checking functions.

The server contains as many routes as devices can be checked. Each route is invoked by the operator to either present the information about a device or as a means to perform a security check on the system. The code Listing 7 shows the server code to handle the request for information about a particular device (e.g., a router) and how the server triggers the security check of its code.

**Listing 7:** Route /dev/router1 tests for a vulnerability in a router device.

@app.route(’/dev/router1’, methods=[’GET’, ’POST’]) **def** r01(): **if** request.method == ’POST’: testList = rrequest.values.getlist("t1") check_list(testList) **return** render_template(’router1.html")

The server returns the client page *router1.html* that allows the operator to select the vulnerabilities to check. The operator selects the particular checks and triggers the test. The test input data provided by the operator (by means of a selection of the form elements of the page) are sent as parameter testList. Function check_list performs the actual check.

This model is flexible as it supports integrating external test code. Most third party codes are based on Python crawlers that are easily combinable with the proposed scheme.

## 4. Implementation and Results

We have performed an experimental validation of the approach by a scale home setting to which we have added the middleware modules that provide the security analysis. In addition, we have performed a performance validation to obtain the temporal behavior of both server and client sides. To test the full capability of the approach, we have integrated a third party testing tool to find vulnerabilities named *wgdscan*; this is added as part of the TEST module, so that it can be easily added and removed from the server functionality bulk.

As explained in Section 1.2, given the integrated nature of our approach in the application level for restricted critical IoT domains, there is no contribution that is comparable to ours. Other contributions found in the state of the art are either focused on network scanning techniques or on mainstream decoupled scanning for general purpose domains. As a result, in the provided implementation, we have undertaken a validation of the approach consisting of demonstrating experimentally that our solution is feasible yielding execution times that do not contravene the operation of the system nor overload the normal operation.

### 4.1. Operation Logging

**Figure 5:**
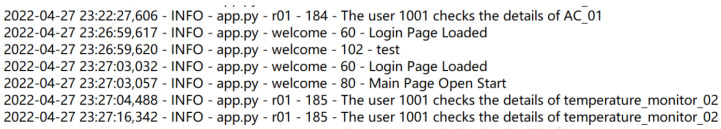
Operations log.

The log functions of Python are used to record the operations in a log that can be later checked for security forensics. This is used at the start of each server function that handled a route, app.logger.info("Log text"). The log text informs about the operation that is performed, e.g., *Operator +* *user* *+ checks info of + device_id*. In addition, the function logging.FileHandler(’test.log’, encoding=’UTF-8’) is used to store all the logs to a particular file (as shown it is file test.log at the server). Figure 5 shows a screenshot of how the log file looks like.

### 4.2. Service Time

The interface module handles the operator HTTP requests to obtain information from the system configuration that comprises the device status and their configuration data.

Requests for device information are formulated to the main server route via a GET method, exhibiting the request parameters as part of the provided URL. This ensures that, on the server-side, there is one route per supported device; this route is generated dynamically. If the development team requires that a particular device is included (or excluded), this can be done as simply as with the activation (or deactivation) of the associated route.

Operator requests to configure devices and other facilities such as login are implemented via POST requests; this includes providing data about the tests. Tests are run though URL crafting with convenient payloads like shown in Table 1. The payload list can be modified at any time as new security issues are either found or discarded.

The validation of our approach requires running a set of experiments to obtain precise temporal costs of the execution of our solution. In order to obtain accurate performance behavior and execution times, the Date class and functions such as Date.getTime have been avoided, as it yields course-grain timing outputs that correspond to mixed states of the web-server and also of the client. The *Navigation Timing* API is used in order to to determine the different *phases* (called *blocks*) undergone by the whole request–response web interaction model. This way, it is possible to extract more accurate measures, and the temporal cost between the occurrence of the different events that takes place in the operation of client and server.

We have used the client side timing API [34] to automate the testing of the web application on the server side. Additionally, we have used Selenium [35] to perform efficient cross-browser tests and further automate the experimental process.

The server side uses Python utilities such as time.perf_counter() that returns the time taken by the execution of a particular function. To obtain a statistical analysis, we use Selenium to automate the process of issuing large numbers of requests.

The server application has been developed in Python, whereas the client side has been developed using mainstream client technology such as HTML, CSS, and JavaScript. The generation of customized client responses has been performed using a templating engine.

**Figure 6:**
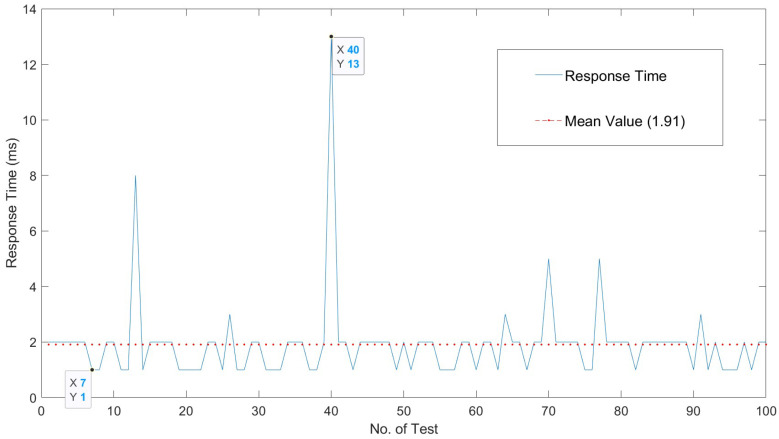
Execution time of response.

**Figure 7:**
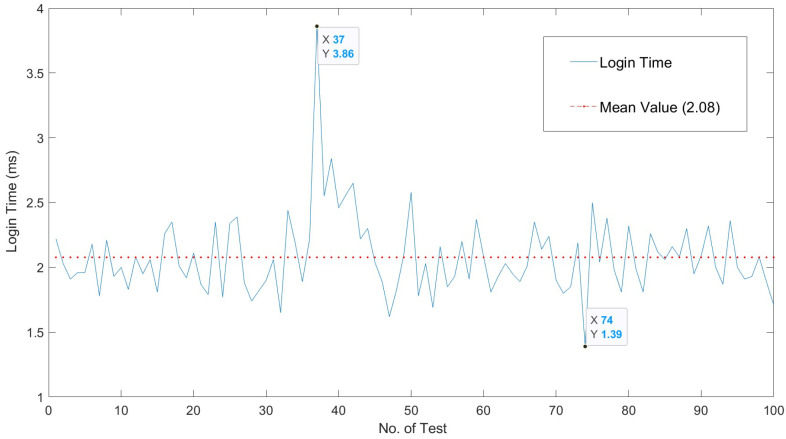
Execution time of login function.

Figure 6 shows the response time of the server in terms of request reception and test execution. Times are measured by taking as base points the loadEventStart and fetchStart of [34]. Obtaining the time in Selenium is done with driver.find_element. A log is created to gather one hundred interactions of ten repetitions each, so that each point that is shown is the average of *n* measures. Figure 7 shows the cost of the server functions involved in the login operation.

**Figure 8:**
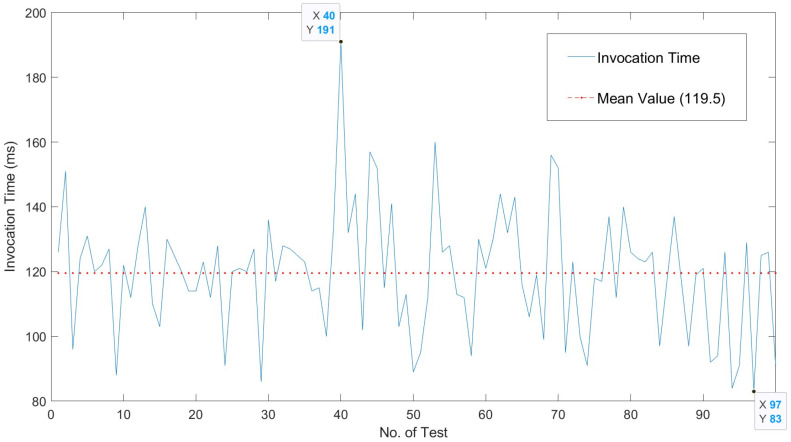
Interaction times.

**Figure 9:**
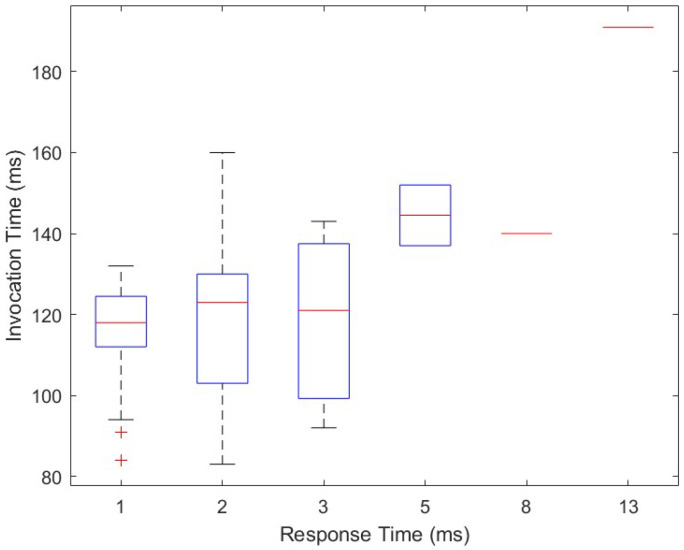
Summary of relation of invocation and response times.

The temporal behavior of our solution is expanded in Figure 8. Figure 8 shows the behavior of our proposed solution when tested to extract the execution times of the whole interaction process between users and server. Ten measures are reported for each experiment. Figure 9 shows the overall invocation time costs in relation to the response times in the extensive testing performed for our solution. The temporal behavior is summarized in Table 2, such that the average interaction time is around 120 ms, with a sustained behavior and a few peaks of 150 ms. The cost of the overall process of request and vulnerability check is low. The overhead of the tests and of the usage of the timing APIs is appropriate to provide a near real-time interaction.

To calculate server processing cost, we instrument it to record start and end time. The start time is the moment of reception of a POST request, whereas the end time is the instant before the next page is loaded.

Responsiveness is one of the major validation criteria for web interaction. A number of studies have appeared over the years that quantify the maximum tolerable waiting times of clients. These numbers take into account a general request–response–display model; however, digging deeper into the computer software execution (from the operating system to the browser), this whole process takes a number of phases or *blocks* that happen at different levels.

According to [36], a response time in the range of 100 to 200 ms is a good response time for a web interaction; being up to 100 ms an optimal interaction time; and above 200 ms up to 1 s an acceptable behavior with room for improvement.

As shown in Figure 6 and Figure 7, the overall execution times are well below this limits; and the overall request–response–display times are in the range of 20% above the limit of what is considered an optimal responsiveness. Consequently, the obtained interaction times correspond to those of a server in the optimal range. Therefore, we can say that our solution provides improved control over vulnerability checking and offers near optimal response times to the user.

## 5. Conclusions

This paper has presented an approach to design and develop a system capable of automatically performing security checks in search of potential vulnerabilities in the IoT nodes and the server. We have shown the workflow of the server to ensure a suitable security level; and have presented a modular design that allows the developer to easily introduce new tests in the server system. To prove the suitability of the approach, we have implemented the server and have performed a set of experiments in order to find the overhead introduced by our deployment. It can be observed that the server performance is stable and that the obtained interaction times allow a near real-time operation with a reduced overhead. Given the power of today’s nodes that run over inexpensive processors that are mostly multicore, it is advisable to perform checks at least periodically. Such nodes may include software pieces like libraries or even microservers and microservices that can include some vulnerabilities. For this purpose, our approach supports testing the vulnerabilities on a per device basis within the system. In addition, we support the easy integration of verified third party vulnerabilities testing tools through the TEST module.

## Figures and Tables

**Table 1:** Payloads used in the tests.

Payload	Type
</script>"><script>prompt(1)</script>	script with prompt box
</ScRiPt>"><ScRiPt>prompt(1)</ScRiPt>	script with prompt box
"><h1 onclick=prompt(1)>Clickme</h1>	HTML tag with prompt box
"><a href=javascript:prompt(1)>Clickme</a>	link to pop-up

**Table 2:** Summary of performance.

Parameter	Value (ms)
Mean	119.5
Maximum	191
Minimum	83

## Data Availability

Not applicable.
